# Assessment of Telemedicine Usage Among Saudis During the COVID-19 Pandemic

**DOI:** 10.7759/cureus.53084

**Published:** 2024-01-27

**Authors:** Asma Alshahrani, Aldanah Alrajhi, Elaf Al Muaythir, Leena Zeyad, Eman M. Mortada

**Affiliations:** 1 Department of Health Sciences, College of Health and Rehabilitation Sciences, Princess Nourah Bint Abdulrahman University, Riyadh, SAU

**Keywords:** saudis, facilitators, barriers, covid-19, telemedicine

## Abstract

Introduction: Telemedicine was the first line of defense during the COVID-19 pandemic. As a result, Saudi Arabia believes it is essential to emphasize telemedicine services to combat the virus as part of its early precautionary actions.

Objectives: To measure the prevalence of telemedicine usage and determine the facilitators and barriers affecting telemedicine usage.

Methods: A cross-sectional study was conducted among Saudis from October 2020 to April 2021. Participants received an online questionnaire through multiple social media platforms.

Results: The prevalence of telemedicine usage was 45.2%. The most significant facilitator that affected the participants' usage was avoiding the infection of COVID-19, at 87.3%. In-person consultation preference was the biggest barrier preventing Saudis from using telemedicine services (62.5%).

Conclusion: Not enough people are aware of telemedicine services, so it is recommended to multiply advertisements for the services provided to reach many users. To enhance Saudi Arabia's awareness of the risks of COVID-19, further studies are needed to assess telemedicine usage during COVID-19, since the majority of studies were conducted in advance.

## Introduction

The World Health Organization (WHO) has identified the coronavirus illness as a pandemic. Due to the infection in several people, it has resulted in a significant number of deaths. The pandemic's growing responsibilities are placing increasing strain on healthcare services. The demand for healthcare services typically rises during pandemics and other public health emergencies, exceeding local capacity [1]. One of the best strategies to slow and stop the spread of the epidemic is through social distance [2]. Telemedicine is an acceptable method to continue providing healthcare to the public, in accordance with Dr. Lurie and Dr. Carr's assessment, which was published in 2018 [3]. Protecting hospital employees and patients from COVID-19 transmission and providing resources, such as staff and personal protection equipment (PPE), to COVID-19 patients are two of the system's key responsibilities during this crisis. To stop the spread of COVID-19, the WHO advised following the following guidelines: lockdown, social withdrawal, hygiene precautions, PPE, isolation, and tracking protocol [4].

The practice of telemedicine first emerged in the middle and late 19th century, but it underwent a major modernization in 1960, principally under the direction of sections of military and space technology. Telemedicine, according to the WHO, is "the delivery of healthcare services, where distance is a critical factor, by all healthcare professionals, using information and communications technologies for the exchange of valid information for the diagnosis, treatment, and prevention of disease and injuries, research and evaluation, and the continuing education of healthcare workers, to advance the health of individuals and communities" [5]. One of the e-health initiatives used in Saudi Arabia in 2010 to improve healthcare services throughout the country was the telemedicine implementation plan, which was completed by the Ministry of Health (MOH) [6].

Globally, China is one of the several nations that have implemented telemedicine. West China Hospital of Sichuan University, provided consultations using the COVID-19 hotline and phone applications. The online consultation system was used by 10179 patients in total from January to March [1]. To increase accessibility, the MOH has implemented the telemedicine system nationally using a hotline number (937), WhatsApp, Twitter, and Instagram. To stop the COVID-19 epidemic, the Saudi Data and Artificial Intelligence Authority and the MOH developed the "Tawakkalna" application. "Tawakkalna" was created to track people's health in terms of possessing COVID-19, being exposed to it, or being immune to it, and reporting to the MOH in cases of exposure or COVID-19 to carry out the policies of the Saudi government. It also requests relocation permits during times of lockdown [7]. Using the MOH phone applications "Seha" and "Mawid," patients can also get in touch with a medical specialist and schedule an appointment at any time [3].

To track citizens' health, the MOH is combining the two apps "Mawid" and "Seha" into one called "Sehhaty." This app makes it simpler for people to schedule appointments and receive their COVID-19 vaccinations by letting them know where the closest vaccination facility is.

It also lets people log their vital signs, physical activity, and other details like height, weight, waist circumference, etc. 160,000 phone calls every week, 3.7 million WhatsApp messages, and 176,000 people use telemedicine services on a monthly basis according to the Mawid application [8]. Saudi Arabia believes it is crucial to focus on telemedicine services to combat the virus as its initial preventative measure [9]. Therefore, it is critical to evaluate how telemedicine was used by Saudis during the COVID-19 pandemic. The main objectives of the current study are to quantify the prevalence of telemedicine usage, to identify the facilitators and barriers affecting telemedicine usage, to report technical issues, doctor-patient communication, and accessibility to telemedicine services, and to assess the level of satisfaction among participants who had used telemedicine during COVID-19 pandemic.

## Materials and methods

Study design, place, and duration

A seven-month cross-sectional study was carried out in Saudi Arabia from October 2020 to April 2021.

Inclusion and exclusion criteria

Being Saudi, older than 18 years old, and agreeing to participate in the study were included. Non-Saudis, those younger than 18 years old, and refused to participate were excluded.

Sampling procedure

Sample Size

The required sample size for the current study is 385 using the online sample size calculator (n4Studies version 1.4.2, Chetta Ngamjarus, Khon Kaen University, Khon Kaen, Thailand). The calculations were based on the assumption that the probability of using telemedicine during the COVID-19 pandemic was 50.0%, at a 95% confidence interval and a limit of precision of 5%, with a design effect of 1.0. The calculated sample size was 385 participants.

Sampling Technique

A non-probability sampling technique was used (the snowball sampling technique) due to the difficulty of reaching the population because of the COVID-19 pandemic.

Data collection tools

Questionnaire

A structured questionnaire that is written in two languages (Arabic and English) and refers to multiple articles [9-13] done on telemedicine. The questionnaire contains six sections with a total of 50 questions. The first section (demographic questions) includes 10 questions (age, gender, marital status, having children, number of children, region, educational level, employment status, family income, and whether they had been infected with COVID-19 or not). The second section is to assess knowledge about telemedicine, which includes four questions. The answers were either "yes, no, I don't know" given a score of 1 for "yes" and 0 for "no" and "I don't know." The score range is between 0 and 4. The participants who had a score between 0-2 are considered to have poor knowledge, while the participants who had a score between 3-4 are considered to have good knowledge. The third section is to assess attitudes toward telemedicine, which includes six questions measured using a five-point Likert scale (5 = strongly agree, 1 = strongly disagree). The score range is between 6 and 30. The participants who had a score between 6-14 are considered to have poor attitudes, while the participants who had a score between 15-30 are considered to have good attitudes. The fourth section is to assess telemedicine usage, which includes four items with a total of 14 questions. The first is the prevalence of telemedicine usage, which contains one question; the answers were scored using (yes = 1, no = 0); the second item is facilitators that encourage telemedicine usage, which consists of six questions; and the third item is barriers to telemedicine usage, which consists of five questions; both items were scored using (1 = yes, 0 = no, 0 = not sure); the fourth item consists of two questions that address the kind of services and type of communication. The total score range is between 0 and 14. The fifth section includes three items (technical issues, doctor-patient communication, and accessibility), with a total of 15 questions and the answers vary from five-point Likert scales (strongly agree = 5, strongly disagree = 1), which consist of nine questions and the score range is 9-45 (always = 4, never = 0) which consist of one question and the score range is 0-4 (yes = 1, no = 0, not sure = 0) which consist of five questions and the range score is 0-5. The sixth section is (patients' satisfaction), which includes three questions, and the answers were scored using a five-point Likert scale (strongly agree = 5, strongly disagree = 1) the score range is 3-15. the participants who had a score between 3-9 are considered not satisfied, while the participants who had a score between 10-15 are considered satisfied.

Data Collection Procedure

The questionnaire went through pilot testing before data collection. We used (Google Forms) to collect the data and distribute it through popular social media channels (WhatsApp, Twitter, etc.). Then the questionnaire was validated by three experts to ensure the acceptability of the questions and the score was 88.5%. The reliability scores were calculated using Cronbach's alpha. For the knowledge section (α = 0.60), attitude section (α = 0.80), facilitators section (α = 0.56), barriers section (α = 0.52), accessibility, doctor-patient communication and technical issues section (α = 0.9), and satisfaction (α = 0.89)

Statistical analysis

The dependent variable was telemedicine usage, while the independent variables were age, gender, marital status, having children, number of children, region, educational level, employment status, family income, COVID-19, level of knowledge, and attitude. Inferential analysis was performed using chi-square, and Fisher exact test when appropriate to compare between participants who use telemedicine and those who do not. Logistic regression was performed to predict significant variables that affect the usage of telemedicine. Data entry and data analysis were done using the JMP® statistical program [14] by SAS Institute Inc., Cary, United States. A p-value of ≤0.05 is considered statistically significant.

Ethical consideration

Ethical approval was obtained by the Institutional Review Board (IRB) of Princess Nourah Bint Abdulrahman University (IRB log number: 20-0514). The informed consent was written in two languages (Arabic and English) and it was obtained from the participants in this research and their registration was anonymous. The privacy and confidentiality of participants' data were protected and will not be used for any other purpose except this study.

## Results

Demographic characteristics

The baseline characteristics of the study participants are shown in Table 1. Of the 385 participants, 214 (55.6%) were females, and 171 (44.4%) were males. The largest age group was 18-34 years (61.3%). The median age of the participants was 27 years, and the interquartile range was 20 years. Of the five regions in Saudi Arabia, the majority of the participants were from the central region. The education level of the participants ranges from the intermediate level or lower (1.6%) to higher education (7.3%), with the largest group being those with a bachelor's degree (65.7%). Most of the participants were students (40%). Regarding the participants' family income, 30.7% have somewhat sufficient income, and 44.1% have sufficient income. For the last variable, the participants were asked whether they previously had or are currently infected with COVID-19 (14.3%) or not (85.7%).

**Table 1:** Demographic characteristics of study participants (n = 385) IQR: Interquartile range

Individual characteristics		Number (n)	Percentage (%)
Age	18-34	236	61.3
	35-49	99	25.7
	50-64	50	13
Age, Median (IQR)	27 (21-40)		
Gender	Male	171	44.4
	Female	214	55.6
Marital status	Not married	218	56.6
	Married	168	44
Having children	Yes	158	41
	No	227	59
Number of children	1-2 children	47	29.8
	3-5 children	75	47.5
	6-8 children	29	18.3
	More than 8 children	7	4.4
Region	Central region	233	60.5
	Northern region	14	3.6
	Southern region	30	7.8
	Eastern region	35	9.1
	Western region	73	19
Educational level	Intermediate level or lower	6	1.6
	Secondary level	98	25.4
	Bachelor	253	65.7
	Higher education	28	7.3
Employment status	Student	154	40.0
	Unemployed	49	12.7
	Government employee	90	23.3
	Private sector employee	41	10.7
	Freelancing	15	3.9
	Retired	36	9.4
Family income	Not sufficient	35	9.1
	Somewhat sufficient	118	30.7
	Sufficient	170	44.1
	More than sufficient	62	16.1
Having COVID-19	Yes	55	14.3
	No	330	85.7

Prevalence of telemedicine usage

Around 45% of the participants had previously used telemedicine services, whereas 55% had never done so.

Facilitators affecting telemedicine usage

**Figure 1:**
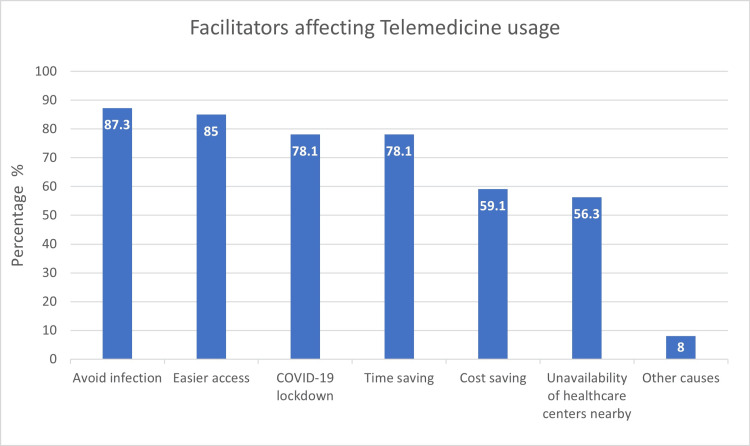
Facilitators affecting telemedicine usage (n = 174)

Easy access to medical services (85%) and preventing infection (87.3%) were the two main facilitators that influenced the participants' use of telemedicine. The absence of nearby healthcare facilities was the least important reason (56.3%). The participants repeated the aforementioned facilitators, as shown in Figure 1 when asked for further justifications.

Barriers limiting telemedicine usage

**Figure 2:**
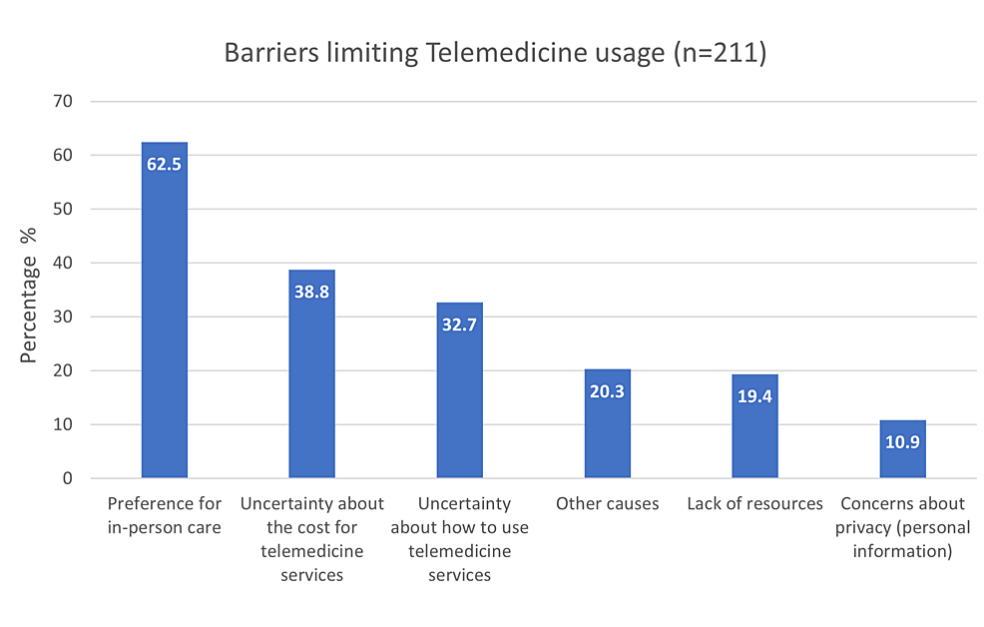
Barriers limiting telemedicine usage (n = 211)

Figure 2 revealed that the preference for in-person care was the highest barrier that limited the participants' telemedicine usage (62.5%). The least contributing barriers were concerns about privacy and personal information (10.9%). Other barriers include the participants' responses, which were classified into three domains: no need to telemedicine (80%), did not know of the service's existence (13.3%), and medical conditions that required in-person care (6.7%), for a total of 30 responses.

Telemedicine usage purpose and type of communication used

The majority of participants have used telemedicine mostly for consultations, while 38% have used it to dispense medication, and 26% have used it for follow-up visits and getting a second opinion from doctors. Only 2.2% of the participants made use of it in other ways. A third of the participants used text messaging, and 68% of the participants used voice calls as their primary form of communication. Email and video calls were used by 14.2% of the participants.

Patients' satisfaction

A greater part of the participants are satisfied with the telemedicine services, only 20% are not satisfied.

Technical issues, doctor-patient communication, and accessibility

Data from participants who had utilized telemedicine are shown in Table 2 regarding technical issues, doctor-patient communication, and accessibility. Regarding the technical difficulties first, 75% of the participants said using telemedicine was simple. The majority of participants agreed that they were able to comprehend the doctor and properly express their health condition throughout their discussion with the doctor. About 78.3% of the participants who made voice or video calls said they could clearly hear the doctor. In terms of accessibility, 74% of the participants concurred that telemedicine access was simple during the COVID-19 epidemic. About 93% of the participants have the tools (phone, computer, internet connection, etc.) needed to access telemedicine. Around 72% of the participants were able to use telemedicine services.

**Table 2:** Technical issues, doctor-patient communication, and accessibility (n = 174)

		Responses	Agree	Neutral	Disagree
Technical issues	The response of the telemedicine service was fast during the COVID-19 pandemic.		119 (68.4%)	35 (20.1%)	20 (11.5%)
	I faced difficulties using telemedicine during the COVID-19 pandemic.		51 (29.3%)	30 (17.2%)	122 (53.5%)
	Generally, the use of telemedicine was easy.		131 (75.3%)	34 (19.5%)	9 (5.2%)
Doctor-patient communication	I was able to hear the doctor clearly when using the voice call or the video call. (Answer the question only if you used a voice or video call.)		123 (78.3%)	23 (14.6%)	11 (7.1%)
	I was able to understand the doctor.		143 (82.2%)	22 (12.6%)	9 (5.2%)
	I was able to explain my condition effectively.		134 (76.8%)	24 (13.8%)	16 (9.4%)
	The doctor's consultation was beneficial.		130 (74.7%)	27 (15.5%)	17 (9.8%)
Accessibility	Access to telemedicine services was easy during the COVID-19 pandemic.		129 (74.1%)	32 (18.4%)	13 (7.5%)

Association between individual characteristics and telemedicine usage

Regarding the association between gender and telemedicine usage, the percentage of females who have used telemedicine is 29.09% and males who have used telemedicine is 16.1%, and with a p-value of 0.002, it suggests that these two percentages are statistically different. The association between telemedicine usage with having children (41.04%) compared to those with no children (58.96%) was statistically highly significant, with a p-value of 0.002. Married participants (43.6%) and not married participants (56.4%) are concluded to be statistically significant by a p-value of 0.039. Meanwhile, the p-value at <0.001 considered highly significant for whether the participants were previously or currently had COVID-19 (14.3%) or have never been infected (85.7%) as revealed in Table 3.

**Table 3:** Association between individual characteristics and telemedicine usage (n = 385)

Individual characteristics		Telemedicine usage		Total	Chi-square test	p-value
		Yes	No			
Age	18-34	97 (25.2%)	139 (36.1%)	236 (61.3%)	4.53	0.103
	35-49	53 (13.8%)	46 (12%)	99 (25.7%)		
	50-64	24 (6.2%)	26 (6.7%)	50 (13%)		
Gender	Male	62 (36.2%)	109 (63.7%)	171 (44.4%)	9.92^a^	0.002*
	Female	112 (52.3%)	102(47.6%)	214 (55.6%)		
Marital status	Not married	88 (22.9%)	129 (33.5%)	217 (56.4%)	4.32	0.039
	Married	86 (21.3%)	82 (23.3%)	168 (43.6%)		
Having children	Yes	86 (22.3%)	72 (18.7%)	158 (41%)	9.22^a^	0.002*
	No	88 (22.9%)	139 (36.1%)	227 (59%)		
Number of children	1-2	25(15.8%)	22 (14%)	47 (29.8%)	3.22	0.359
	3-5	41 (26%)	35 (21.5%)	75 (47.5%)		
	6-8	15 (9.5%)	14 (8.9%)	29 (18.4%)		
	More then 8	6 (3.8%)	1 (0.6%)	7 (4.4%)		
Region	Central	113(29.4%)	120 (31.2%)	233 (60.6%)	4.98	0.289
	Northern	3 (0.78%)	11 (2.9%)	14 (3.6%)		
	Southern	12(3.1%)	18(4.7%)	30 (7.8%)		
	Eastern	14 (3.6%)	21 (5.5%)	35 (9.1%)		
	Western	32(8.3%)	41 (10.6%)	73 (18.9%)		
Educational level	Intermediate	4 (1%)	2 (0.5%)	6 (1.5%)	1.60	0.659
	Secondary	42 (10.9%)	56 (14.6%)	98 (25.5%)		
	Bachelor	114 (29.6%)	139 (36.1%)	253 (65.7%)		
	Higher	14 (3.6%)	14 (3.6%)	28 (7.2%)		
Employment status	Student	68(17.7%)	86 (22.3%)	154 (40%)	2.42	0.788
	Unemployed	20 (5.2%)	29 (7.5%)	49 (12.7%)		
	Government	46 (12%)	44 (11.4%)	90 (23.4%)		
	Private sector	16 (4.2%)	25 (6.5%)	41 (10.7%)		
	Freelancing	7 (1.8%)	8 (2.1%)	15 (3.9%)		
	Retired	17 (4.4%)	19 (4.9%)	36 (9.3%)		
Family income	Not sufficient	11 (2.8%)	24 (6.2%)	35 (9%)	3.40	0.333
	Somewhat sufficient	52 (13.5%)	66 (17.1%)	118 (30.7%)		
	Sufficient	81 (21%)	89 (23.1%)	170 (44.2%)		
	More than sufficient	30 (7.7%)	32 (8.3%)	62 (16.1%)		
Having COVID-19	Yes	40 (10.4%)	15 (3.9%)	55 (14.3%)	19.63^a^	<0.001*
	No	134 (34.8%)	196 (50.9%)	330 (85.7%)		
^*P value ≤ 0.05 is significance^ ^; a: Calculated using Fisher test^						

Association between level of knowledge, attitude, and telemedicine usage

There were 36.2% of participants with a high degree of knowledge who also used telemedicine services, as opposed to 8.57% of those with a low level of expertise. The p-value for this difference is less than <0.001, making it statistically very significant. There was a significant difference in people's attitudes concerning telemedicine services between those who had favorable attitudes (37.4%) and those who had negative attitudes (17.4%), with a p-value of 0.002 (Table 4).

**Table 4:** Association between level of knowledge, attitude, and telemedicine usage (n = 385)

		Telemedicine usage		Total	Chi-square test	p-value
		Yes	No			
Level of knowledge	Good	141 (36.6%)	123 (32%)	264 (68.6%)	22.8^a^	<0.001*
	Poor	33 (8.5%)	88 (22.9%)	121 (31.4%)		
Attitude	Positive	143 (37.1%)	144 (37.4%)	287 (74.5%)	9.76^a^	0.002*
	Negative	31 (8.1%)	67 (17.4%)	98 (25.5%)		
^*p-value ≤ 0.05 is significance;^ ^a: Calculated using Fisher test^						

Predictable variables of telemedicine usage

According to binary logistic regression analysis, the variables strongly predict Saudi Arabians' use of telemedicine during the COVID-19 epidemic. The best predictor of telemedicine use was having COVID-19 (OR: 4.396, 95% CI: 2.246-8.602, p = 0.001). While the amount of knowledge also indicates a high probability of using telemedicine during the COVID-19 pandemic (OR: 0.345, 95% CI: 0.209-0.570, p = 0.001) (Table 5).

**Table 5:** Logistic regression for variables predicting telemedicine usage among Saudis during the COVID-19 pandemic (n = 385)

Variable	B	Wald	p-value	Exp(B)	95% CI for Exp(B)	
					Upper	Lower
Gender	0.521	5.125	0.024*	1.685	1.072	2.647
Having children	0.538	5.516	0.019*	1.712	1.093	2.684
Having COVID-19	1.480	18.694	<0.001*	4.396	2.246	8.602
Level of knowledge	-1.061	17.277	<0.001*	0.345	0.209	0.570
Attitude	-0.758	8.021	0.005*	0.468	0.277	0.791
Constant	-0.427	3.975	0.46	0.652		
^*p-value ≤ 0.05 is significant; B: Unstandardized beta regression coefficient^						

## Discussion

To maintain social distancing and deliver medical care during the COVID-19 pandemic, health practitioners used telemedicine. Telemedicine was emphasized by Saudi Arabia as a means of controlling the coronavirus's spread. The objectives of the current study are to measure the prevalence of telemedicine service usage and to determine the facilitators and barriers affecting telemedicine service usage. Also, to report the technical issues, communication with the doctor, and accessibility of telemedicine services. In addition to evaluating patient satisfaction with telemedicine services among Saudis. This was done by conducting a cross-sectional study with a sample size of 385 among the citizens of Saudi Arabia.

In COVID-19, 45.2% of the participants used telemedicine, which is in line with a poll of 2000 Americans, the results of which also revealed that 42% of respondents used telemedicine to combat the pandemic [15]. Telemedicine services were in more demand as a result of the outbreak and lockdown, which is a clear indication of how widely used telemedicine is becoming. On the other hand, a case report analysis using data gathered from the main New York University healthcare system records revealed a striking increase in the prevalence rate of 2000% when comparing video visits to the average day before the pandemic.

Achieving over 7,000 visits in just 10 days, which is also accounted for by the quick onset of the pandemic, necessitated the urgent use of online consultations like video visits [16]. While being able to be compared to the findings of the current investigation, the data from NYU Langone Health Center is not genuinely representative. Instead of focusing on the number of users, it is providing information on the prevalence of virtual visits to healthcare systems.

It was not surprising that during the pandemic, participants most frequently used telemedicine because they worried about contracting the disease (87%) and because it made accessing healthcare services easier (85%). It is obvious why most people would want a simpler and safer manner of providing healthcare, especially during a pandemic that affects the entire world. At the time this study was being written, however, there was no literature evaluating these telemedicine utilization facilitators. The second explanation, improved access to healthcare services, can be attributable to two factors. First, the lockdown that took place at the start of the outbreak restricted participants' access to the delivery of their regular healthcare. The Saudi 2030 Vision is the second justification, which led to the MOH expanding its telemedicine services in 2018 [17]. Around 56% of telemedicine users claimed that the absence of nearby healthcare options was the reason they used the service. The factors described above can be blamed for this, even if it is the least frequent factor that led to the use of telemedicine.

In the current study, 62% of the participants indicated a preference for in-person care. Convenience, affordability, and time savings are factors that explain their preference for online consultation over in-person consultation, even though a prior survey conducted in the UK indicated that 18% of participants chose in-person consultation [18]. The utilization of the telemedicine service was unclear to almost one-third of the participants. In contrast to other research in a systematic review, which also noted telemedicine's limited use as a barrier related to ambiguity about how to use it [19]. Only a small percentage of the participants in this study who did not use telemedicine expressed privacy concerns about the system. The use of telemedicine services is restricted by privacy, according to certain articles in another systematic review [20]. The cost of telemedicine services was unclear to nearly 39% of research participants.

Around 44% of participants expressed uncertainty regarding the cost, which is consistent with findings from another study. Most of the time, when using telemedicine services, both patients and doctors are not informed of the cost of the service, and this could be a reason for not using telemedicine services [21]; some study participants do not have the required resources (mobile, laptop, etc.) to use telemedicine services. Similar to this, in a prior fast review research, the absence of devices and internet connections may restrict the use of telemedicine services [22]; In another systematic review, a few studies cited a lack of adequate devices as a hindrance to telemedicine use [20]. In the current study, 7.4% of all forms of communication included video calls, which were thought to be quite low. However, retrospective cohort research carried out in New York City found that 21.88% of the participants made use of video calls when using the telemedicine service. Saudis are more conservative, as was expected; therefore, this variation in percentages may be due to cultural differences [23].

A study using mixed methods on a sample of Bangladeshis found that nearly half of the participants believed telemedicine services were available around the clock [11]. The majority of Saudi respondents to the current study gave an indistinguishable response. The majority of participants did not have any problems using telemedicine. Similar to this, most of the participants in a different study reported no technical problems [24]. Most participants concurred that they could understand the doctor's explanation of their problem, which was done straightforwardly and concisely. The participants' responses to questions concerning their virtual consultation were consistent with those of another study carried out in the United States [25].

The doctor could be understood clearly by more than half of the participants. In contrast, research conducted in Japan found that just a small portion of participants could comprehend the doctor clearly [26]. This survey's findings, which show that 74.1% of participants thought telemedicine was easily accessible, are congruent with those of a similar study done in the United States, which found that the majority of participants thought telemedicine was simple to use [25]. Saudi Arabia is ranked number 33 out of 79 countries in Huawei's Global Connectivity Index because of its exceptional broadband capacity, which offers quick internet and, in turn, benefits access to telemedicine.

This study demonstrates several strengths. The researchers achieved a substantial sample size, with 45% of participants reporting telemedicine usage, aligning with similar studies conducted in the United States. This prevalence rate highlights the global adoption of telemedicine as a response to the pandemic as well as the correspondence between telemedicine use between culturally different states. Furthermore, the findings shed light on the impact of the Saudi MOH's efforts to expand telemedicine services across the Kingdom as part of the Saudi 2030 Vision. This strategic initiative, combined with the lockdown measures, resulted in increased telemedicine usage among participants who faced limited access to traditional healthcare services. However, we must acknowledge the limitation associated with using a non-probability sampling technique is selection bias. This might have affected the generalizability of the findings, by creating a smaller pool of participants that is not representative of the population meant to use telemedicine services. In addition, exploring the causes behind the unsatisfactory responses would offer further insight into what essentially could be a barrier to the use of telemedicine. Even though the MOH stated that their e-health services would cover all areas of the Kingdom, our study was not able to reach the Saudi population living in rural areas. Finally, as a result of the novelty of the topic during the first COVID-19 pandemic, insufficient studies were conducted about telemedicine services during the COVID-19 pandemic globally and in Saudi Arabia especially.

## Conclusions

This study evaluated Saudi Arabians' use of telemedicine during the COVID-19 pandemic. About half of the Saudis in the tested population claimed to have used telemedicine during the COVID-19 pandemic. The primary driver for the respondents' usage of telemedicine was their desire to avoid catching COVID-19, whereas the primary barrier was their preference for in-person consultations. Nonetheless, among the sample, it was discovered that gender, having children, having COVID-19, knowledge, and attitude were predictors of telemedicine use. Because not enough people are aware of telemedicine services, it is advised to increase the number of advertisements for the services offered to reach a large user base. To encourage health providers to suggest telemedicine services to their patients with low medical priority and to decrease the proportion of people who prefer in-person consultations. The MOH has to do more to inform the public about the e-health services offered and should also offer instructional videos or infographics on how to use these services. Since the majority of studies were carried out before COVID-19, additional research is also required to evaluate the use of telemedicine during this crisis.
